# N-terminal region of *Saccharomyces cerevisiae *eRF3 is essential for the functioning of the eRF1/eRF3 complex beyond translation termination

**DOI:** 10.1186/1471-2199-7-34

**Published:** 2006-10-11

**Authors:** Valery N Urakov, Igor A Valouev, Natalia V Kochneva-Pervukhova, Anna N Packeiser, Alexander Yu Vishnevsky, Oleg O Glebov, Vladimir N Smirnov, Michael D Ter-Avanesyan

**Affiliations:** 1 Institute of Experimental Cardiology, Cardiology Research Center, Moscow, 121552, Russia; 2 Shemyakin-Ovchinnikov Institute of Bioorganic Chemistry RAS, Moscow, 117997, Russia; 3 MRC Laboratory of Molecular Biology, Cambridge, CB2 2QH, UK

## Abstract

**Background:**

Termination of translation in eukaryotes requires two release factors, eRF1, which recognizes all three nonsense codons and facilitates release of the nascent polypeptide chain, and eRF3 stimulating translation termination in a GTP-depended manner. eRF3 from different organisms possess a highly conservative C region (eRF3C), which is responsible for the function in translation termination, and almost always contain the N-terminal extension, which is inessential and vary both in structure and length. In the yeast *Saccharomyces cerevisiae *the N-terminal region of eRF3 is responsible for conversion of this protein into the aggregated and functionally inactive prion form.

**Results:**

Here, we examined functional importance of the N-terminal region of a non-prion form of yeast eRF3. The screen for mutations which are lethal in combination with the *SUP35-C *allele encoding eRF3C revealed the *sup45 *mutations which alter the N-terminal domain of eRF1 and increase nonsense codon readthrough. However, further analysis showed that synthetic lethality was not caused by the increased levels of nonsense codon readthrough. Dominant mutations in *SUP35-C *were obtained and characterized, which remove its synthetic lethality with the identified *sup45 *mutations, thus indicating that synthetic lethality was not due to a disruption of interaction with proteins that bind to this eRF3 region.

**Conclusion:**

These and other data demonstrate that the N-terminal region of eRF3 is involved both in modulation of the efficiency of translation termination and functioning of the eRF1/eRF3 complex outside of translation termination.

## Background

Termination of protein synthesis takes place in ribosomes in a response to stop codons in the "decoding" site. The proteins that control this process are called polypeptide chain release factors (RFs). In eubacteria there are two class 1 polypeptide chain release factors, RF1 and RF2, recognizing UAA/UAG and UAA/UGA stop codons, respectively, whereas eukaryotes employ only one such factor, eRF1, which is able to decode all three nonsense codons [1]. In addition, translation termination in eubacteria and eukaryotes is stimulated by class 2 release factors, RF3 and eRF3, respectively. Both class 2 factors are GTPases enhancing the termination efficiency by stimulating activity of class 1 release factors in a GTP-dependent manner [2-4]. RF3 is able to remove RF1 or RF2 from the ribosome after hydrolysis of the ester bond in peptidyl tRNA, i.e. to stimulate recycling of class 1 factors [5]. In contrast to eubacterial RF3, eukaryotic eRF3 functions in termination by applying its GTPase activity to assist eRF1 with the stop codon recognition and/or ensures rapid and efficient hydrolysis of peptidyl tRNA [6]. Importantly, this was shown upon the excess of eRF1 when quantitative peptide release did not require recycling of eRF1. In the yeast *Saccharomyces cerevisiae *eRF1 and eRF3 are encoded by the essential *SUP45 *and *SUP35 *genes, and often designated as Sup45p and Sup35p, respectively [3].

Members of the eRF1 family have been identified in a number of eukaryotes and they all show a high degree of sequence similarity. Determination of the crystal structure of human eRF1 has shown that it is composed of three domains and resembles by overall shape and dimensions a tRNA molecule with the N-terminal and middle domains corresponding to the tRNA's anticodon stem and aminoacyl acceptor stem, respectively [7]. eRF3 also has a complex structure and can be divided into at least two regions: a non-conserved N-terminal region and a conserved C-terminal region, showing a considerable sequence similarity to the translation elongation factor eEF1A, which brings aminoacyl-tRNAs to the ribosomal A site. The C-terminal part (domain C) of the *S. cerevisiae *eRF3 is responsible for the function of the protein in translation termination. The N-terminal region of yeast eRF3 is non-essential for the translation termination and viability and may be further subdivided into the N-terminal (N) domain, which was shown to be required for maintenance of the cytoplasmically-inherited prion determinant [*PSI*^+^] [8,9] and the middle (M) domain, which also is important for [*PSI*^+^] propagation [10,11]. The nonsense suppressor phenotype of [*PSI*^+^] reflects eRF3 aggregation and functional inactivation [12,13]. Thus, the N domain of eRF3 may inhibit translation termination in *S. cerevisiae *in a [*PSI*^+^] prion-dependent manner. However, other data have shown that this domain also affects function of a non-prion form of yeast eRF3, since deletion of the eRF3 N-terminal extension (N and M domains) decreased nonsense codon readthrough caused by depletion of eRF1 [14]. This agrees with the results showing that N-terminal extension of the *Schizosaccharomyces pombe *eRF3 may inhibit translation termination by blocking eRF1 binding to eRF3 [15]. However, the N-terminal region of eRF3 from the filamentous fungus *Podospora anserina *confers quite opposite effect on translation termination, since deletion of this protein region caused suppression of nonsense mutations [16]. The effects of N-terminal truncation of eRF3 have a species-specific character probably because of the lack of sequence similarity between the N-terminal regions of eRF3 homologs from different organisms. Nevertheless, despite of structural dissimilarity, the N-terminal regions of eRF3 from yeast and mammals are able to interact with poly(A)^+ ^binding protein PABP, which probably serves for coupling termination and initiation steps of translation [17,18] and for regulation of mRNA deadenylation and decay [19]. The N-terminal region of *S. cerevisiae *eRF3 also is able to bind other proteins, such as Itt1p, a protein with unknown function, and Sla1p assisting in the cytoskeleton assembly [20,21]. These interactions may modulate translation termination efficiency and link translation with other cellular processes.

Biochemical studies of *S. cerevisiae *eRF3 are hampered by its propensity to aggregate [22,23]. This makes genetic approach preferable to investigate function of this release factor. Here, we identified mutations which alter eRF1 and make the N-terminal region of eRF3 indispensable. The study of these mutations and their lethal interaction with the *SUP35 *deletion allele, encoding the eRF3 C-terminal domain, indicate the role of N-terminal region of this factor in non-translational function of the eRF1/eRF3 termination complex in yeast.

## Results

### Identification and characterization of mutants in which deletion of the eRF3 NM region is lethal

The *SUP35-C *strain YJV159-rS35C carrying the pHT-SUP35 (*SUP35 ADE3*) centromeric plasmid was mutagenized with UV irradiation and over 150,000 of grown colonies were screened using a yeast colony color sectoring assay. Among them, 23 were of Sect^- ^phenotype. Such clones could arise due to mutations in the *SUP35-C *gene, since it is essential [8], or due to mutations in other genes. To discriminate between these possibilities, the centromeric pRS315-SUP35C (*SUP35-C LEU2*) plasmid was introduced into the YJV159-rS35C strain carrying pHT-SUP35. In 22 cases this restored Sect^+ ^phenotype indicating that mutations were within *SUP35-C *and corresponding mutants were discarded. One mutant, which segregated white sectors very poorly, preserved its leaky Sect^- ^phenotype in the presence of the pRS315-SUP35C plasmid. The mutant, designated as YJV-rS35C-SL23, could not grow at 37°C on YPD plate. This mutant carrying pHT-SUP35 was transformed with a genomic library based on the *LEU2 *centromeric plasmid p366, and two transformants able to grow at non-permissive temperature were selected. The obtained temperature resistant transformants were characterized by the frequent loss of pHT-SUP35 at permissive temperature, thus being Sect^+^. Plasmid from one transformant was isolated and partial sequencing of the cloned DNA fragment has shown that it contained the *SUP45 *gene. Subsequent delimitation of the functional region of the isolated DNA fragment indicated that *SUP45 *was responsible for both temperature resistance and Sect^+ ^phenotype. Transformation of this mutant with various *SUP45*-carrying plasmids caused the same effects. Thus, mutation that made the NM region of eRF3 indispensable arose in the *SUP45 *gene. Transformation of the mutant YJV-rS35C-SL23 carrying pHT-SUP35 with the multicopy plasmid YEplac181-SUP35NM encoding the eRF3 NM region did not restore the Sect^+ ^phenotype. This indicated that expression of the entire eRF3 protein is necessary for viability of this mutant.

**Figure 1:**
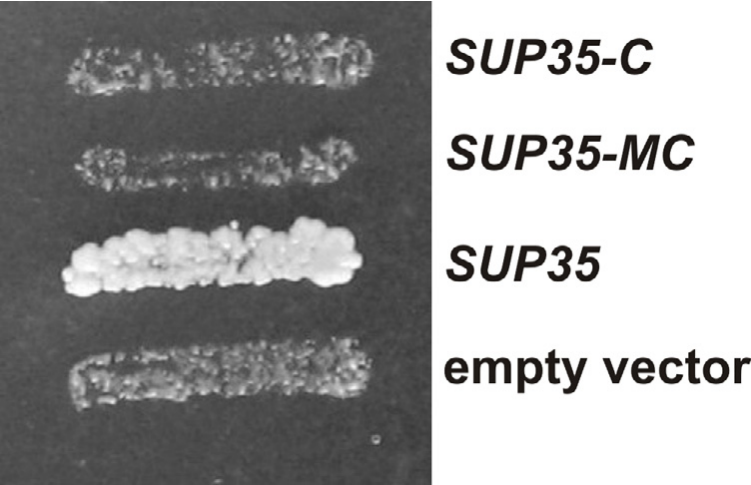
The *sup45-sl23*^*ts *^mutation is lethal in the strain 33G-D373-rSL23-ΔS35, carrying the *SUP35-C *or *SUP35-MC *alleles. The *LEU2 *plasmids with *SUP35-C *or *SUP35-MC *can not replace the resident *URA3 *plasmid pRS316-SUP35 carrying wild type *SUP35 *in the strain 33G-D373-rSL23-ΔS35. Transformants were incubated on 5FOA-containing medium for 3 days. Plasmids: *SUP35-C*, pRS315-SUP35C; *SUP35-MC*, pRS315-SUP35MC; *SUP35*, pRS315-SUP35; empty vector, pRS315.

The obtained *sup45 *mutation, designated as *sup45-sl23*^*ts*^, caused growth inhibition in the strain YJV-rS35C-SL23, but was lethal in other *sup45-sl23*^*ts *^strains with the *SUP35*-C allele. For example, the *URA3 SUP35 *plasmid pRS316-SUP35 of the *SUP35*-disrupted strain 33G-D373-rSL23-ΔS35 carrying *sup45-sl23*^*ts *^could not be replaced with the *LEU2 SUP35-C *plasmids upon incubation on 5FOA medium (Fig. 1). It is also important that in this strain the plasmid pRS316-SUP35 also could not be replaced with the centromeric pRS315-SUP35MC plasmid, encoding eRF3 which lacks only the N domain (Fig. 1). Thus, just the absence of the eRF3 prion-forming domain was responsible for synthetic lethality.

Synthetic lethality was also reproduced in the S1-R-H8 strain. This strain, carrying *SUP35-C*, was disrupted for *SUP45 *and contained the *URA3 SUP45 *centromeric plasmid pRG415-SUP45, which could not be replaced with the plasmid pRS315-sup45-sl23 carrying the mutant *sup45-sl23*^*ts *^allele. In contrast, in the control strain S1-H8 with chromosomal wild type *SUP35*, pRS315-sup45-sl23 could replace the resident pRG415-SUP45 plasmid. The lethal effect of combination with *SUP35-C *was also found for another *sup45 *mutation, *sup45-36*^*ts *^[24]: the pRS315-sup45-36 plasmid did not replace pRG415-SUP45 in the S1-R-H8 strain, but replaced this plasmid in the *SUP45 *disruptant S1-H8 bearing the wild type *SUP35 *gene.

The possible reason of lethality manifested by the *sup45 *mutations in combination with *SUP35-C *is the decreased level of eRF1. However, the amount of eRF1 was not affected in the *sup45-36*^*ts *^[25] and *sup45-sl23*^*ts *^mutants (data not shown). *S. cerevisiae *eRF1 and eRF3 interact with each other through the binding sites located at their C-termini [26,27], though the eRF3 N-terminal region also may be involved in this interaction [28]. Mutations in eRF1 breaking its interaction with eRF3 decrease cell viability [27]. The observed disparity in genetic interaction of the *sup45-sl23*^*ts *^and *sup45-36*^*ts *^mutations with *SUP35 *and *SUP35-C *suggests that these mutations influence binding of eRF1 to eRF3, and this effect is more pronounced when eRF3 lacks its N-terminal region. However, the study of the ability of *Escherichia coli*-expressed His_6_-eRF1 and His_6_-eRF1-sl23 immobilized on TALON beads to precipitate eRF3 and eRF3C from yeast cell lysates [27] did not show a noticeable effect of *sup45-sl23*^*ts *^mutation on the interaction of eRF1 with both eRF3 and eRF3C (data not shown).

**Figure 2:**
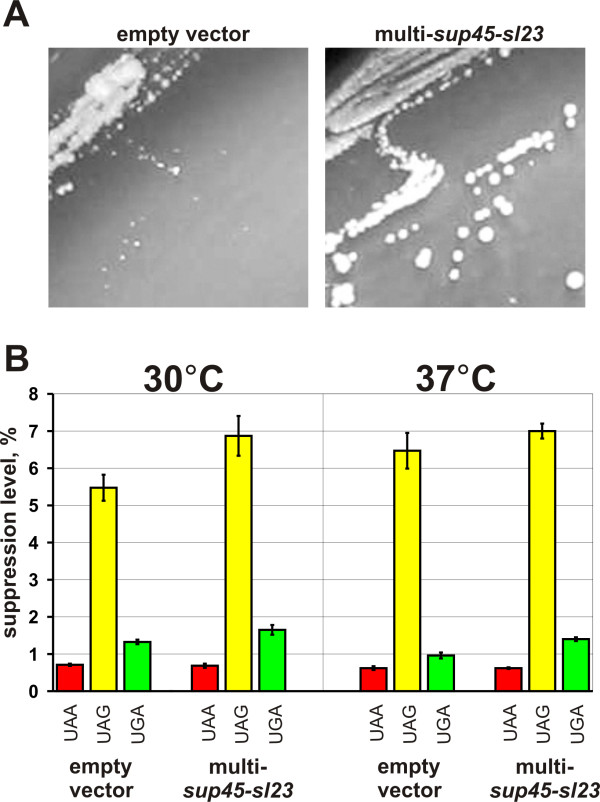
Extra copies of *sup45-sl23*^*ts *^improve growth of the strain 33G-D373-rSL23 carrying the chromosomal *sup45-sl23*^*ts *^mutation at 37°C (A), but do not influence nonsense codon readthrough (B). Empty vector, YEplac181; multi-*sup45-sl23*, YEplac181-sup45-sl23. Photos were taken after incubation of transformants on leucine omission medium for 4 days. The strain 33G-D373-rSL23 carrying the plasmids indicated above, was transformed with either one of the pUKC815, 817, 818, 819 plasmids and grown in medium selective for plasmids to mid log phase. Then appropriate aliquots of yeast culture were taken and β-galactosidase activity was assayed. Suppression efficiency (average from three independent transformants, each in three parallels) was estimated as described [44].

**Figure 3:**
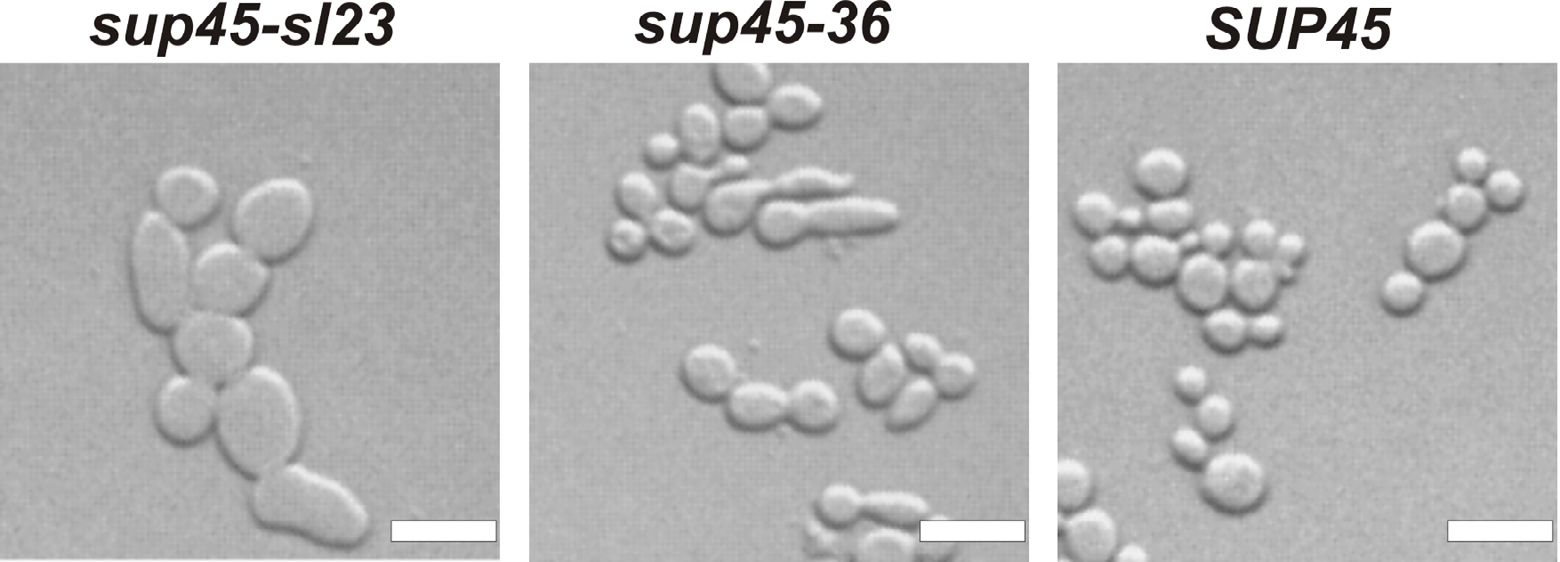
Cell morphology defects of the *sup45 *mutants incubated at 37°C. The strain 33G-D373-ΔS45 disrupted for *SUP45 *and bearing the plasmids pRS315-sup45-sl23 (*sup45-sl23*), pRS315-sup45-36 (*sup45-36*) or pRS315-SUP45 (*SUP45*), was grown in liquid YPD medium for 15 h at 37°C. Bar = 6 μm.

The *sup45-sl23*^*ts *^and *sup45-36*^*ts *^mutations altered the eRF1 N domain (Ser-30 to Phe and Leu-34 to Ser substitutions, respectively), increased nonsense codon readthrough and caused temperature sensitivity [25]. At elevated temperature corresponding mutations did not cause any noticeable increase in the levels of nonsense codon readthrough (Fig. 2 and data not shown), but affected cell morphology. The *sup45-36*^*ts *^mutation caused defect of cytokinesis and resulted in appearance of multinucleated cells with elongated buds. The defect of cytokinesis in this mutant is related to mislocalization of the myosin light chain Mlc1p and its temperature sensitivity could be suppressed by Mlc1p overproduction [25]. In contrast, temperature sensitivity of the *sup45-sl23*^*ts *^mutant could not be alleviated by excess of Mlc1p. Cells of the *sup45-sl23*^*ts *^mutant were increased in size (Fig. 3) and contained single nuclei (data not shown). Temperature sensitivity of the *sup45-sl23*^*ts *^mutant can not be explained by a defect of translation termination, since increase of the number of *sup45-sl23*^*ts *^copies allowed mutant to grow at 37°C, but did not decrease the levels of nonsense codon readthrough either at permissive or non-permissive temperature (Fig. 2). It is also noteworthy that extra copies of the *sup45-sl23*^*ts *^allele did not restore viability of the strain YJV-rS35C-SL23 carrying chromosomal *sup45-sl23*^*ts *^and *SUP35-C*, indicating that temperature sensitivity and synthetic lethality were caused by the impairment of different cellular processes.

### Mutations in SUP35-C abolishing its synthetic lethality with sup45-sl23^ts ^or sup45-36^ts^

Mutations in *SUP35-C *which make it compatible with *sup45-sl23*^*ts *^were obtained by passing the corresponding plasmid in the *E. coli *"mutator" strain. Five independent pools of the mutagenized plasmid were obtained and used to transform the strain YJV-rS35C-SL23 which carried the chromosomal *sup45-sl23*^*ts *^and *SUP35-C *alleles and the centromeric *SUP35 ADE3 *pHT-SUP35 plasmid. Over 100,000 of transformed cells were incubated on leucine omission medium at 37°C and 116 Leu^+ ^temperature resistant clones were selected. Streaking of these transformants on leucine omission medium showed that 75 of them produced colonies with Sect^+ ^phenotype. The plasmid DNA from 8 Sect^+ ^transformants was isolated and used for repeated transformation of the strain YJV-rS35C-SL23 carrying pHT-SUP35. All Leu^+ ^transformants bearing the isolated plasmids were temperature resistant and able to lose the resident pHT-SUP35 (*SUP35 ADE3 URA3*) plasmid on medium supplemented with uracil, suggesting that they carried mutant *SUP35-C*. It is noteworthy that, since these mutations were dominant, they restored the normal growth rate and temperature resistance of the *sup45-sl23*^*ts *^mutant YJV-rS35C-SL23 carrying the chromosomal *SUP35-C *allele. These *SUP35-C *mutant alleles supported viability of the *sup45-sl23*^*ts *^mutant 33G-D373-rSL23-ΔS35 disrupted for *SUP35 *and allowed its growth at non-permissive temperature, indicating that suppression of temperature sensitivity did not depend on genetic background.

Sequencing of the mutant *SUP35-C *alleles revealed that they had nucleotide substitutions that caused either Glu-539 to Lys (five cases, isolated from four mutagenized plasmid pools), Ala-596 to Val (two cases, both derived from the same plasmid pool) or Ala-597 to Val (one case) replacements. It is noteworthy that these amino acid substitutions occurred in the positions located downstream to the eRF3 regions implicated in GTP hydrolysis [3,8]. The corresponding alleles were respectively designated as *SUP35-C14*, *SUP35-C11 *and *SUP35-C42*.

**Figure 4:**
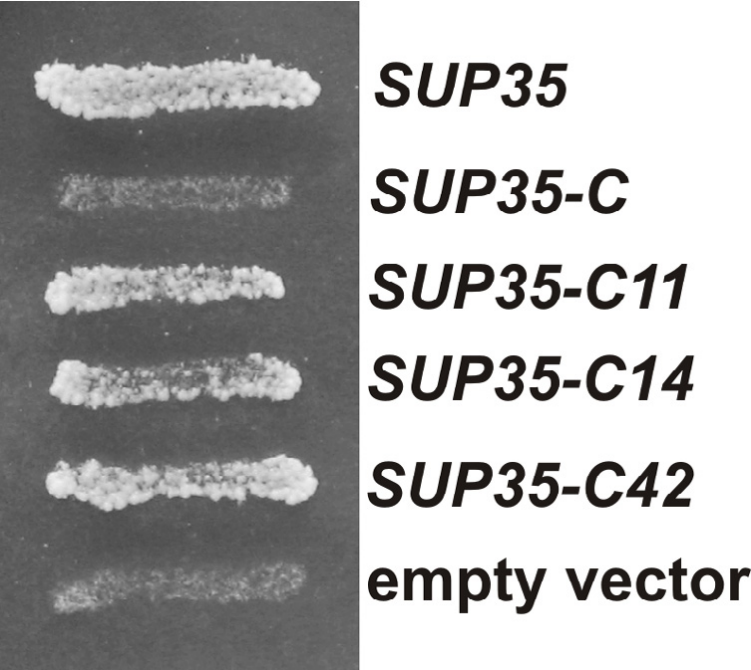
Compensatory mutations in plasmid *SUP35-C *restore viability of the strain carrying the *sup45-36*^*ts *^and *SUP35-C *alleles. The *sup45-36*^*ts *^mutant 33G-D373-r36-ΔS35 disrupted for *SUP35 *and carrying the *SUP35 URA3 *pRS316-SUP35 plasmid was transformed with either one of the *LEU2 *plasmids pRS315-SUP35 (*SUP35*), pRS315-SUP35C (*SUP35-C*), pRS315-SUP35-C11 (*SUP35-C11*), pRS315-SUP35-C14 (*SUP35-C14*), pRS315-SUP35-C42 (*SUP35-C42*) or pRS315 (empty vector). Transformants were incubated on 5FOA-containing medium for 3 days.

The isolated compensatory *SUP35-C *mutations were studied for the ability to alleviate temperature sensitivity caused by the *sup45-36*^*ts *^mutant allele as well as to suppress its synthetic lethality with *SUP35-C*. Introduction of either one of the plasmids with the *SUP35-C14*, *SUP35-C11 *or *SUP35-C42 *alleles into the *sup45-36*^*ts *^mutant 33G-D373-r36-ΔS35, which was disrupted for *SUP35 *and carried the pRG415-SUP35 (*URA3 SUP35*) plasmid, allowed the loss of the resident plasmid on 5-FOA-containing medium at permissive temperature (Fig. 4), but did not cause resistance to 37°C. Thus, the compensatory mutations suppressed lethality caused by the combination of either *sup45-sl23*^*ts *^or *sup45-36*^*ts *^with *SUP35-C*, but did not suppress temperature sensitivity of *sup45-36*^*ts*^.

### Synthetic lethality between sup45-sl23^ts ^or sup45-36^ts ^and SUP35-C is not caused by the increased levels of nonsense codon readthrough

**Figure 5:**
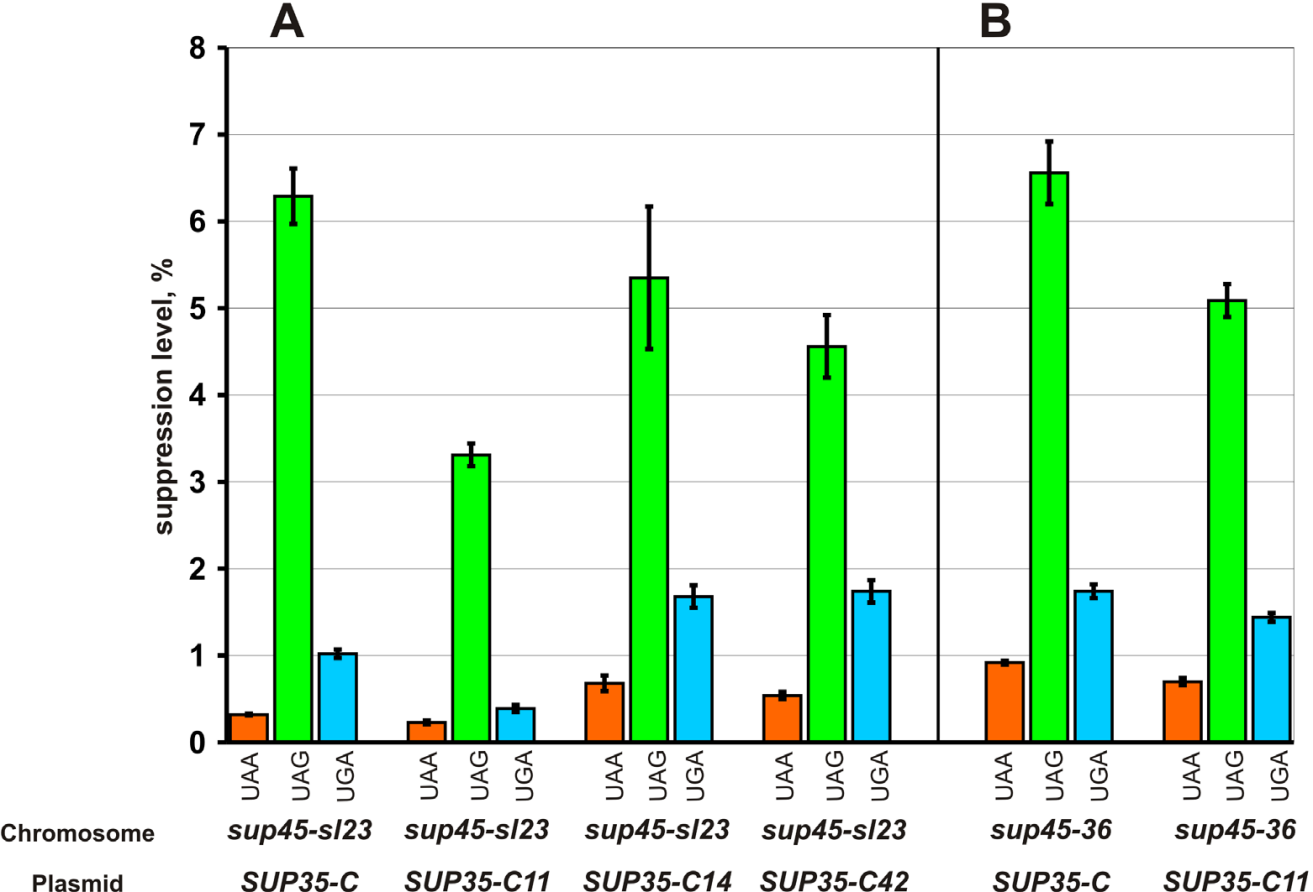
Influence of the compensatory mutations in *SUP35-C *on the levels of nonsense codon readthrough in the *sup45-sl23*^*ts *^33G-D373-rSL23 (A) and *sup45-36*^*ts *^33G-D373-r36 (B) mutants. Plasmids: *SUP35-C*, pRS315-SUP35C; *SUP35-C11*, pRS315-SUP35C11; *SUP35-C14*, pRS315-SUP35C14; *SUP35-C42*, pRS315-SUP35C42. Suppression efficiency (average from three independent transformants, each in three parallels) was estimated with the use of the pUKC815, 817, 818, 819 plasmids.

We also tested, whether the *SUP35-C *compensatory mutations influenced the nonsense codon readthrough caused by the *sup45-sl23*^*ts *^and *sup45-36*^*ts *^mutations. For this, the strain 33G-D373-rSL23 carrying the chromosomal *sup45-sl23*^*ts *^mutation and wild type *SUP35*, was transformed with centromeric plasmids containing either the *SUP35-C *allele (control) or alleles with the *SUP35-C14*, *SUP35-C11 *or *SUP35-C42 *compensatory mutations along with either one of the pUKC815, 817, 818, 819 plasmids. Quantitative assay demonstrated that the nonsense codon readthrough was noticeably decreased only in the presence of *SUP35-C11*. This compensatory mutation also decreased the levels of nonsense readthrough in the *sup45-36*^*ts *^mutant 33G-D373-r36 carrying wild type *SUP35 *but to a lesser extent than in the isogenic *sup45-sl23*^*ts *^mutant 33G-D373-rSL23 (Fig. 5). Thus, though the effect of *SUP35-C *compensatory mutations on the levels of nonsense readthrough was not significant in most cases, they all abolished synthetic lethality, making unlikely that the increased levels of readthrough was the reason of lethal interaction between the *sup45*^*ts *^mutations and *SUP35*-C allele.

To confirm this we have constructed the strains 33G-D373-rSL23-r35C and 33G-D373-r36-r35C, which carried, respectively, the *sup45-sl23*^*ts *^and *sup45-36*^*ts *^mutations and the *SUP35-C *allele, as well as the centromeric pCM183-SUP45 plasmid with the *TRP1 *selectable marker and *SUP45 *under the control of regulatable *tetO*_2 _promoter. The synthetic lethality of *sup45*^*ts *^and *SUP35-C *combination in these strains was manifested only upon the repression of wild type *SUP45 *by doxycycline. As a control we used the isogenic strains 33G-D373-rSL23 and 33G-D373-r36, which had the wild type *SUP35 *gene instead of the *SUP35-C *allele. To monitor the levels of nonsense codon readthrough upon the repression of wild type *SUP45*, all strains were transformed with either one of the pUKC815, 817, 818, 819 plasmids.

**Figure 6:**
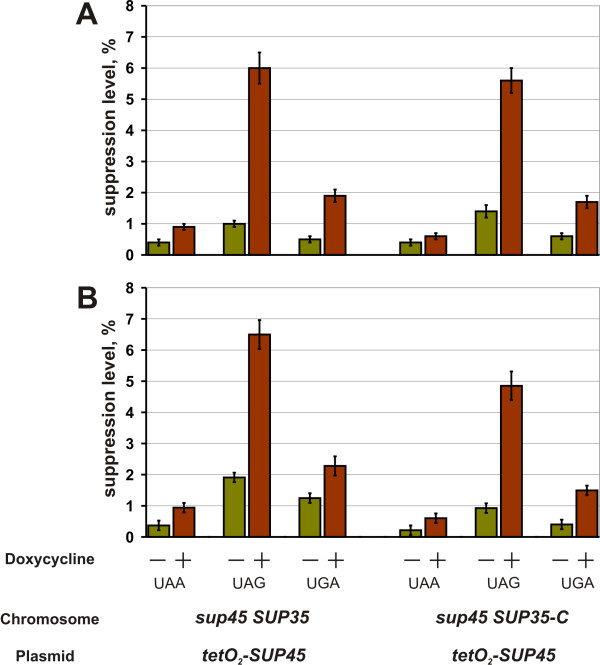
Interaction between the *sup45*^*ts *^and *SUP35-C *alleles does not result in increase of the efficiency of nonsense codon readthrough. Data for the 33G-D373-rSL23 (*sup45-sl23*^*ts *^*SUP35*) and 33G-D373-rSL23-r35C (*sup45-sl23*^*ts *^*SUP35-C*) strains (A), as well as for 33G-D373-r36 (*sup45-36*^*ts *^*SUP35*) and 33G-D373-r36-r35C (*sup45-36*^*ts *^*SUP35-C*) strains (B) are presented. All strains carrying the pCM183-SUP45 plasmid (*tetO*_2_*-SUP45*) were transformed with either one of the pUKC815, 817, 818, 819 plasmids and grown in tryptophane omission medium to mid log phase. Then the cells were collected, resuspended in analogous medium containing 10 μg/ml doxycycline. After 17 h of incubation in this medium, appropriate aliquots of yeast culture were taken and β-galactosidase activity was assayed. All data represent an average of at least three independent experiments.

Since chromosomally-encoded mutant eRF1 and its wild type counterpart encoded by the plasmid were electrophoretically indistinguishable, we could not estimate a decline in the amount of eRF1 upon the repression of the plasmid *SUP45 *in the studied strains. However, in another strain with the same *tetO*_2_*-SUP45 *plasmid, 17 h repression decreased the amount of eRF1 to approximately 5% of its initial levels. Importantly, this drop in wild type eRF1 amount was accompanied with enrichment of cultures with dead cells up to 20% for the 33G-D373-rSL23-r35C and 33G-D373-r36-r35C strains, while the number of such cells in the 33G-D373-rSL23 and 33G-D373-r36 cultures was less than 10%. The *in vivo *quantification of UAA, UAG, and UGA nonsense readthrough has shown that its levels in the strains with synthetic lethal mutations was not higher than in the control strains (Fig. 6). This disagrees with the assumption that the cause of synthetic lethality between *sup45 *mutations and *SUP35-C *allele is too high levels of nonsense codon readthrough. Incubation of the strain 33G-D373-rSL23-r35C carrying the *tetO*_2_*-SUP45 *plasmid in medium containing doxycycline did not cause any change in the polyribosome content or distribution (data not shown), indicating absence of the sharp effect on translation.

## Discussion

Here, we have demonstrated that the *sup45-sl23*^*ts *^mutation did not decrease the levels of eRF1, and *sup45-sl23*^*ts *^extra copies neither suppressed synthetic lethality, nor decreased nonsense codon readthrough. This indicates that both defect of translation termination and lethal interaction between *sup45-sl23*^*ts *^and *SUP35-C *were not due to a shortage of eRF1, making improbable suggestion that these effects were due to inefficient recycling of mutant eRF1 by eRF3 lacking the N-terminal region. Furthermore, such suggestion contradicts to our previous data indicating that the NM region of eRF3 may negatively affect its ability to recycle eRF1. It was shown that shortage of eRF1 in the strain expressing full length eRF3 resulted in higher levels of nonsense codon readthrough than in the strain expressing eRF3C [14], while deletion of the eRF3 NM region did not affect nonsense readthrough at the normal levels of eRF1 [examined in the presence of the tRNA suppressor *SUP16 *(*SUQ5*) to enable detection of a decrease in nonsense readthrough levels; our unpublished data]. This suggests that the presence of eRF3 NM region has a significant negative effect on translation termination only at the decreased eRF1 levels. Deficiency in eRF1 should make its recycling important for translation termination and, therefore, these data agreed with the hypothesis that eRF3 is involved in the recycling of eRF1 and indicated that NM region of eRF3 interferes with this process. Thus, deletion of NM region should enhance the eRF3 activity, making improbable that the reason of synthetic lethal interaction between *SUP35-C *and *sup45-sl23*^*ts *^or *sup45-36*^*ts *^lies in inefficient translation termination. Furthermore, the results of this work suggest that combination of *SUP35-C *with either *sup45-sl23*^*ts *^or *sup45-36*^*ts *^is lethal not due to an increase of nonsense codon readthrough.

The obtained *SUP35-C *mutations compensate for the lack of the eRF3 N-terminal region, thus ruling out the possibility that synthetic lethality was due to a disruption of interaction with proteins that bind to this eRF3 region, including interaction with the poly(A)^+^-binding protein PABP [18,19]. This makes unlikely that synthetic lethality was caused by a disturbance in regulation of mRNA decay or by alteration of translation initiation by its uncoupling from termination. The latter is also supported by the observation that synthetic lethality was not accompanied by noticeable alteration of the polyribosomal profile.

Both identified *sup45 *mutations, *sup45-sl23*^*ts *^and *sup45-36*^*ts*^, that make the N-terminal region of eRF3 indispensable, altered the N domain of eRF1 and, furthermore, they caused substitutions of amino acids located in proximity to each other. Both *sup45-sl23*^*ts *^and *sup45-36*^*ts *^increased the levels of nonsense codon readthrough and, therefore, affected translation termination. In addition, they did not allow yeast cells to grow at 37°C. Earlier it was found that temperature sensitivity of the *sup45-36*^*ts *^mutant was not due to a defect of translation termination. Inability to grow at non-permissive temperature was caused by the impairment of cytokinesis resulting from mislocalization of the eRF1-binding protein, myosin light chain Mlc1p [25]. Though cytokinesis defect was not shown for the *sup45-sl23*^*ts *^mutant, its temperature sensitivity also was not caused by a defect in translation termination, suggesting that yeast eRF1 may have different functions outside of translation. It is also important that the compensatory *SUP35-C *mutations alleviated both effects of the *sup45-sl23*^*ts *^mutation, temperature sensitivity and increased nonsense readthrough, indicating that interaction with eRF3 probably is important for eRF1 not only for promoting translation termination, but also for its uncharacterized function. In contrast, interaction with eRF3 is not required for function of eRF1 in cytokinesis, since eRF1 in a complex with Mlc1p can not bind eRF3 [25].

## Conclusion

The data presented here show that the effect of synthetic lethality can not be deduced from a defect of translation termination. The study of the *SUP35-C *compensatory mutations has shown that they are able to suppress both temperature sensitivity of *sup45-sl23*^*ts *^and its lethal interaction with *SUP35-C*. However, temperature sensitivity of *sup45-sl23*^*ts *^and its synthetic lethality with *SUP35-C *are caused by different reasons, since increasing the copy-number of *sup45-sl23*^*ts *^alleviated temperature sensitivity but did not abolish synthetic lethality. Therefore, these data show that the eRF1/eRF3 complex may be involved in different processes outside of translation termination.

In many yeast species the N-terminal part of the eRF3 release factors is responsible for their prion properties [29-33]. Such conservation suggests that the prion properties of yeast eRF3 have biological significance. In this work we have shown that the prion-forming domain of eRF3 from the yeast *S. cerevisiae *is important for its functioning outside of translation termination. However, this does not mean that the N-terminal extension of eRF3 factors from other organisms plays a similar role. Indeed, point mutations altering *S. cerevisiae *eRF3C can compensate for the lack of its NM region indicating that the C domain of eRF3 from other organisms may contain all information required for this function.

## Methods

### Media and strains

Yeast strains were grown on standard organic (YPD) and synthetic (SD) media [34]. The modified YPD media (YPDm) [2% peptone, 0.5% yeast extract (Oxoid), 3% glucose and 2% agar] was used in colony color sectoring assay experiments, because it favored accumulation of red pigment in the *ade2 *mutants. The 5-fluoroorotic acid (5FOA) medium was prepared as described [35]. The expression of the *tetO*_2-_controlled *SUP45 *was repressed by incubation of corresponding strains on medium which contained 10 μg/ml doxycycline and was selective for the pCM183-SUP45 plasmid. LB and 2 × YT media were used for bacteria [36]. Appropriate amounts of antibiotics, amino acids, and bases were added when necessary. Yeast cells were grown at 30°C, if not indicated otherwise, and bacteria at 37°C. DNA transformation of lithium acetate-treated yeast cells was performed as described previously [37]. *E. coli *cells were transformed by the method described in [38].

The *E. coli *mutator strain XL1-Red [*endA*1 *gyrA*96 *thi-*1 *hsdR*17 *supE*44 *relA*1 *lac mutD*5 *mutS mutT *Tn10 (Tet^r^)] (Stratagene) was used for obtaining mutations in the plasmid *SUP35-C *gene. For this, the plasmid pRS315-SUP35C was introduced into this *E. coli *strain. After incubation for 48 h, over 500 transformant colonies were suspended in 10 ml of 2 × YT supplemented with ampicillin (100 μg ml^-1^). Cell culture was additionally incubated for 8 h with shaking, plasmid DNA was isolated and used to transform yeast. The *E. coli *strain DH5α [*supE*44 D*lac *U169 (f80 *lacZ*DM15) *hsdR*17 *recA*1 *endA*1 *gyrA*96 *thi*-1 *relA*1] [36] was used in cloning experiments.

The yeast strains used are listed along with their genotypes in Table 1. Construction of strains obtained in this study is described below. The strain YJV159-rS35C, which contains the 5'-deletion *SUP35-C *allele (encoding the eRF3 fragment lacking amino acids 1-253, eRF3C) in place of the wild-type *SUP35 *allele, was obtained as follows. The pFL44-SUP35C integrative plasmid [8] bearing the *SUP35-C *allele was linearized by *Sal*I site internal to the *SUP35 *coding sequence and integrated into the *SUP35 *gene of the strain YJV159 by selecting transformants on uracil omission medium. The Ura^+ ^integrants expressing both eRF3 and eRF3C were obtained and placed onto 5FOA-containing medium to select for plasmid excision. The obtained Ura^- ^clones were screened by Western blotting to select strains expressing eRF3C only. Replacement of *SUP35 *with *SUP35-C *was further confirmed by Southern analysis. The same integration/excision method was used to obtain the temperature-sensitive *sup45 *mutants 33G-D373-rSL23 (*sup45-sl23*^*ts*^) and 33G-D373-r36 (*sup45-36*^*ts*^). The integrative plasmids pJJ244-sup45-sl23 and pJJ244-sup45-36 bearing the *sup45-sl23*^*ts *^and *sup45-36*^*ts *^alleles, respectively, were linearized by *Msc*I and integrated into the chromosomal *SUP45 *gene of the strain 33G-D373. The obtained Ura^+ ^transformants were placed on 5FOA-containing medium for plasmid excision and Ura^- ^clones with the suppressor (growth on tryptophane and histidine omission media) and temperature sensitive (inability to grow on YPD at 37°C) phenotypes were selected. Then, the obtained strains 33G-D373-rSL23 and 33G-D373-r36 were disrupted for *SUP35 *to obtain respectively the strains 33G-D373-rSL23-ΔS35 and 33G-D373-r36-ΔS35. To perform this, the *Xho*I-*Ppu*10I DNA fragment of the plasmid pSUP35::TRP1 was used to transform the strains 33G-D373-rSL23 and 33G-D373-r36 harboring the pRS316-SUP35 (*SUP35 URA3*) plasmid. The clones unable to grow on 5FOA plates (without pRS316-SUP35) were selected. Disruption of *SUP35 *in these strains was confirmed by PCR analysis. The strains 33G-D373-rSL23-rS35C (*sup45-sl23*^*ts*^*SUP35-C*) and 33G-D373-r36-rS35C (*sup45-36*^*ts*^*SUP35-C*) were constructed in the same way as YJV159-rS35C. However, the pRS315-SUP45 (*SUP45 LEU2*) plasmid was introduced into the 33G-D373-rSL23 and 33G-D373-r36 strains before the integration/excision procedure to prevent synthetic lethality of the *sup45*^*ts *^and *SUP35-C *alleles. The pRS315-SUP45 plasmid was then replaced with the pCM183-SUP45 (*tetO*_2_*-SUP45 TRP1*) plasmid.

### Plasmids

The plasmids used are listed along with their essential characteristics in Table 2. The pRS315-SUP35, pRS316-SUP35, pHT-SUP35 and pSUP35::TRP1 plasmids were constructed as follows. The *SUP35*-containing *Xho*I-*Xba*I DNA fragment of the plasmid pEMBLyex4-SUP35 [8] was inserted into the *Xba*I-*Sal*I sites of pRS315, pRS316, pET-30a(+) (Novagen) and pBluescript II KS(+) (Stratagene) vectors giving the plasmids pRS315-SUP35, pRS316-SUP35, pET-S35 and pBS-SUP35, respectively. Next, the *Xba*I-*Not*I DNA fragment of pET-S35 containing *SUP35 *was cloned into the same sites of the centromeric *ADE3 URA3 *vector pHT4467 [39] resulting in pHT-SUP35. The *TRP1*-containing *Acc*65I-*Bgl*II DNA fragment of the pJJ281 plasmid [40] was cloned into the *Bsr*GI and *Bcl*I sites of pBS-SUP35. *Hpa*I-*Hpa*I DNA fragment of the resulting plasmid was deleted, and thus, the plasmid pSUP35::TRP1 used for disruption of the chromosomal *SUP35 *gene was constructed. The *Mlu*I/Klenow-*Xba*I DNA fragment of the plasmid pEMBLyex4-SUP35C containing the 5'-deletion *SUP35-C *allele [8] was inserted between the *Sma*I and *Xba*I sites of pRS315 and as a result the plasmid pRS315-SUP35C was obtained. The plasmid pRS315-SUP35MC was constructed by the insertion of *Pvu*II-*Xba*I DNA fragment of pEMBLyex4-SUP35MC [8] which contained the mutant *SUP35-MC *allele encoding eRF3 without the N domain into the *Sma*I and *Xba*I sites of pRS315. Mutant alleles *SUP35-C11*, *SUP35-C14 *and *SUP35-C42*, cloned into the shuttle vector pRS315, were sequenced using a set of primers specific for *SUP35*.

The *SUP45 *gene was cloned using a yeast genomic library based on the plasmid p366, which was identical to YCp50 [41], but had *LEU2 *instead of *URA3 *(gift of P. Hieter). Isolation and sequencing of the *sup45-sl23*^*ts *^allele was described earlier [25]. The plasmid YEplac181-sup45-sl23 was obtained by insertion of the *Xba*I-*Xho*I DNA fragment of pRS315-sup45-sl23 containing the mutant *sup45-sl23*^*ts *^allele into the *Xba*I and *Sal*I sites of YEplac181. The *Xho*I-*Sac*I DNA fragments carrying the *sup45 *mutant alleles of the plasmids pRS315-sup45-36 and pRS315-sup45-sl23 were cloned between the *Sal*I and *Sac*I sites of the integrative *URA3 *vector pJJ244 [40] to obtain the plasmids pJJ244-sup45-36 and pJJ244-sup45-sl23, respectively. The pCM183-SUP45 plasmid bearing the *SUP45 *gene under the control of regulatable *tetO*_2 _promoter was constructed as follows. Using the plasmid pRS315-SUP45 as template, PCR was applied to place the *Nco*I site (CCATGG) at the *SUP45 *translation initiation codon. The obtained PCR fragment was digested with *Nco*I and *Xho*I and cloned into the same sites of the *E. coli *plasmid pET-30a(+) (Novagen) to generate the plasmid pL51-3. The *Kpn*I-*Xho*I fragment of pL51-3, containing the coding sequence of *SUP45*, was inserted into the *Kpn*I and *Sal*I sites of YEplac112 resulting in the YEplac112-atgSUP45 plasmid. Then, the *Eco*ICRI-*Pst*I fragment of YEplac112-atgSUP45 was cloned into the *Hpa*I and *Pst*I sites of pCM183 [42].

β-galactosidase activity in cell lysates was quantified as described [43]. The levels of UAA, UAG and UGA readthrough were determined using the pUKC815/817/818/819 series vectors, as described previously [44].

### Colony color sectoring assay

The assay is based on the facts that *ade2 *yeast cells accumulate a red pigment and form red colonies but *ade2 ade3 *cells do not accumulate this pigment and, consequently, form white colonies because the *ade3 *mutation blocks the pathway at a point prior to the accumulation of the pigment [45]. Thus, a strain that is *ade2 ade3*, but carries *ADE3 *on an autonomously replicating plasmid, forms on medium non-selective for this plasmid red colonies (cells with the plasmid) that contain white sectors (cells that have lost the plasmid). We refer this phenotype as Sect^+ ^(ability to lose the *ADE3 *plasmid), and, consequently, the phenotype of red clones without white sectors or with a negligible number of such sectors is Sect^- ^(inability to lose the *ADE3 *plasmid). Multiple suspensions of the *SUP35-C ade2 ade3 *strain YJV159-rS35C carrying the centromeric plasmid pHT-SUP35 (*SUP35 ADE3*) were prepared in liquid uracil omission medium; each suspension deriving from a separate colony grown on uracil omission medium was diluted, and plated on ten YPDm plates at approximately 5,000 cells per plate. These cells were mutagenized with UV irradiation to approximately 10% survival and then incubated for 4 or 5 days. As expected, most clones were Sect^+^. Cells from Sect^- ^colonies were streaked on YPDm and after 2 days, individual colonies were restreaked on YPDm. Only strains that continued to give red colonies or red colonies with rare white sectors were studied further.

## Authors' contributions

VU, performed a screen for the synthetic lethal mutations and participated in molecular genetic studies, IV carried out the molecular genetic studies and drafted the manuscript, NK-P, AP, AV and OG carried out the molecular genetic studies, VS participated in planning of experiments and drafted the manuscript, MT-A designed the study and wrote the manuscript. All authors read and approved the final manuscript.

## Figures and Tables

**Table 1:** *S cerevisiae *strains used in this study

Strain	Genotype	Source/reference
YJV159	*MAT*a *ade2 ade3 his3 leu2-3,112 trp1 ura3*	[46]
YJV159-rS35C	Same as in YJV159, but with *SUP35-C *instead of *SUP35*	This study
YJV159-rS35C-SL23	Same as in YJV159-rS35C, but with *sup45-sl23*^*ts *^instead of *SUP45*	This study
33G-D373	*MAT*a *ura3-52 leu2-3 112 trp1-289 his7-1 lys9-A21 pheA10 ade2-144, 717*	[47]
33G-D373-rSL23	Same as in 33G-D373, but with *sup45-sl23*^*ts *^instead of *SUP45*	This study
33G-D373-rSL23-rS35C	Same as 33G-D373-rSL23 but with *SUP35-C *instead of *SUP35*	This study
33G-D373-r36	Same as in 33G-D373, but with *sup45-36*^*ts *^instead of *SUP45*	This study
33G-D373-r36-rS35C	Same as 33G-D373-r36 but with *SUP35-C *instead of *SUP35*	This study
33G-D373-rSL23-ΔS35	Same as in 33G-D373-rSL23, but with *sup35::TRP1*	This study
33G-D373-r36-ΔS35	Same as in 33G-D373-r36, but with *sup35::TRP1*	This study
33G-D373-ΔS45	Same as in 33G-D373, but with *sup45::ADE2*	[25]
S1-H8	*MAT*a *leu2-3,112 his3-*Δ*1 ura3-52 trp1-289 sup45::TRP1*	[14]
S1-R-H8	Same as in s1-H8, but with *SUP35-C *instead of *SUP35*	[14]

*his7-1 *and *lys9-A21 *and *ade2-1*, ochre mutations, *trp1-289*, amber mutation.

**Table 2:** Plasmids used in this study

Plasmid	Characteristics	Source/reference
YEplac181	Multicopy *LEU2 *vector	[48]
pRS315	Centromeric *LEU2 *vector	[49]
pRS316	Centromeric *URA3 *vector	[49]
pRG415-SUP45	Centromeric *URA3 *vector with *SUP45*	[14]
pRS315-SUP45	Same as pRS315, but with *SUP45*	[25]
pRS315-sup45-36	Same as pRS315, but with the *sup45-36*^*ts *^mutant allele	[25]
pRS315-sup45-sl23	Same as pRS315, but with the *sup45-sl23*^*ts *^mutant allele	[25]
YEplac181-sup45-sl23	Same as YEplac181, but the *sup45-sl23*^*ts *^mutant allele	This study
pRS315-SUP35	Same as pRS315, but with *SUP35*	This study
pRS315-SUP35-C11	Same as pRS315, but with *SUP35-C11*	This study
pRS315-SUP35-C14	Same as pRS315, but with *SUP35-C14*	This study
pRS315-SUP35-C42	Same as pRS315, but with *SUP35-C42*	This study
pRS316-SUP35	Same as pRS316, but with *SUP35*	This study
pFL44-SUP35C	Integrative *URA3 *vector with *SUP35-C*	[8]
pSUP35::TRP1	Integrative vector with the *sup35::TRP1 *disruption allele	This study
pHT-SUP35	Centromeric *ADE3 URA3 *vector containing *SUP35*	This study
pRS315-SUP35C	Same as pRS315, but with *SUP35-C*	This study
pRS315-SUP35MC	Same as pRS315, but with *SUP35-MC*	This study
pJJ244-sup45-sl23	Integrative *URA3 *vector with the *sup45-sl23*^*ts *^allele	This study
pJJ244-sup45-36	Integrative *URA3 *vector with the *sup45-36*^*ts *^allele	This study
pCM183-SUP45	Centromeric *TRP1 *vector containing *SUP45 *under the control of regulatable *tetO*_2 _promoter	This study
